# Metals and metalloids concentrations in adults aged 50 and older from Mexico

**DOI:** 10.21203/rs.3.rs-8107374/v1

**Published:** 2025-12-01

**Authors:** David Hernández-Bonilla, Marlene Cortez-Lugo, Victor Hugo Ríos Baza, Horacio Riojas-Rodríguez, Halle Cathey, Rebeca Wong

**Affiliations:** National Institute of Public Health: Instituto Nacional de Salud Publica; National Institute of Public Health; National Institute of Ecology and Climate Change; National Institute of Public Health: Instituto Nacional de Salud Publica; National Institute of Public Health: Instituto Nacional de Salud Publica; National Institute of Public Health: Instituto Nacional de Salud Publica

**Keywords:** Metal exposure, Older adults, Hair biomarkers, Sociodemographic factors

## Abstract

**Objectives.:**

Quantify concentrations of metals/metalloids in hair samples from Mexican adults aged 50 years and older and analyze their relationship with sociodemographic characteristics.

**Methods.:**

A cross-sectional study with 2,474 participants from the 2018 Mexican Health and Aging. Metals/metalloids in hair samples were analyzed using inductively coupled plasma mass spectrometry and optical emission spectroscopy. Concentrations were compared by sex, age, locality size, educational attainment, marital status, and socioeconomic status, using nonparametric statistical methods.

**Results.:**

Lead, titanium, manganese, and copper were detected in most samples (≥95%), with copper exhibiting the highest median concentration (7.83 μg/g). Differences by sex were observed in 13 elements: males showed higher concentrations, except for copper and titanium, which were higher in females. Increasing age was associated with lower concentrations of copper, manganese, nickel, titanium, and vanadium. In contrast, higher education and a middle-to-high socioeconomic status were linked with increased concentrations of several elements; notably, manganese concentrations were highest among those with low socioeconomic status. Locality size showed minimal effects, except for slightly higher manganese concentrations in urban participants. Regarding marital status, individuals who were married or in a consensual union displayed higher concentrations of chromium, manganese, nickel, and lead.

**Conclusions.:**

This study provides reference values for exposure to metals and metalloids in older Mexican adults, highlighting sociodemographic patterns of accumulation. Age, sex, education, socioeconomic status, and marital status were relevant factors. Findings underscore the need for population-based biomonitoring, additional research on the health impacts, and targeted public health interventions.

## 1. Introduction

Biomarkers of exposure measure the internal dose of environmental pollutants in individuals and are essential tools for studying the public health effects of contamination (Bergdahl and Skerfving 2008; Fowler 2009; Zoni and Lucchini 2013; Arora et al. 2014; Haynes et al. 2015; Coetzee et al. 2016; Santos Serrão de Castro and de Oliveira Lima 2018; García-Chimalpopoca et al. 2019). Hair matrices have gained recognition as biomarkers of metal and metalloid exposure because metals are incorporated into hair through systemic circulation during follicle formation (Benderli Cihan and Oztürk Yıldırım 2011; Chanpiwat et al. 2015).

While biomarkers of exposure in conventional biological matrices—including blood, urine, and bone tissue—are widely accepted indicators for assessing exposure to metals and metalloids like lead (Pb), mercury (Hg), and arsenic (As), these have facilitated the establishment of associations between exposures and public health risks (Chan et al. 2020; Zhang et al. 2023). However, hair offers unique analytical advantages as a biomarker: it is non-invasive, easily collected, transported, and stored without special requirements. Additionally, hair samples provide a retrospective exposure window, reflecting cumulative exposure to elements over the past 1–2 months (Chojnacka et al. 2012; Coetzee et al. 2016; Jiang et al. 2017; Liang et al. 2017).

Although hair matrices are a valuable biomarker for epidemiological studies, there are potential disadvantages. In particular, hair is susceptible to contamination from external sources, including dust, water, and hair products.^3^ Therefore, proper sample cleaning and measurement techniques are crucial to estimate endogenous exposure (Dongarrà et al. 2011; Hanh et al. 2011; Hernández-Bonilla et al. 2011; Eastman et al. 2013; Menezes-Filho et al. 2014; Oulhote et al. 2014; Lucas et al. 2015; Haynes et al. 2015; Skröder et al. 2017). Some studies have shown significant variations in hair metal/metalloid concentrations. Whether these variations are due to differences in the cleaning effectiveness or reflect actual differences in exposure remains unclear (Stauber and Florence 1989; Razagui 2008; Eastman et al. 2013).

Various factors affect the concentration of metals/metalloids in human hair. Environmental pollution varies depending on whether a person lives in an urban or rural area and their proximity to exposure sites, such as industrial or mining locations. Additional sources of exposure include air, soil, water, food, occupation, and lifestyle choices like smoking (Briggs 2003). This study aims to quantify the concentrations of metals/metalloids in the hair of individuals aged 50 and older who participated in the fifth wave of the Mexican Health and Aging Study (MHAS) in 2018, and to analyze how sociodemographic factors may be linked to differences in exposure.

## 2. Materials and methods

### 2.1 Study design and population

This cross-sectional study analyzes a randomized subsample of 3,000 Mexican adults aged 50 and older from the fifth wave of the MHAS in 2018. The MHAS is a nationally representative longitudinal study that focuses on aging, health, mortality, and disability in Mexican adults aged 50 and older. Launched in 2001, it has followed participants through seven waves, introducing new cohorts in 2012, 2018, and 2024. Study participants gave informed consent before completing the survey and giving hair samples. The Institutional Review Boards approved the MHAS protocols at the University of Texas Medical Branch (USA), the National Institute of Statistics and Geography (Mexico), and the National Institute of Public Health (Mexico).

### 2.2 Hair metals/metalloids

#### 2.2.1. Hair sample collection, preservation, and handling

**Figure 1:**
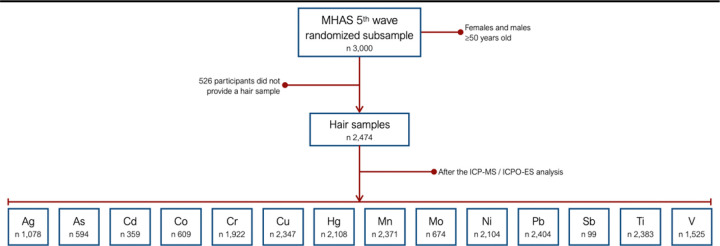
Hair samples from 2018 MHAS

During the 2018 MHAS interview, trained interviewers from the National Institute of Statistics and Geography collected hair samples from 2,474 participants who consented to donate their hair (Fig. 1). Each sample, weighing approximately 3 g, was obtained from the occipital region of the scalp, with the hair growth pole and the end closest to the scalp clearly identified. The samples were carefully labeled, preserved in plastic bags, and stored at 4°C for transport and later analysis at the National Institute of Ecology and Climate Change laboratory in Mexico.

#### 2.2.2. Hair metal/metalloid analysis

To eliminate potential contaminants, 300 mg of hair was initially washed with 100 mL of acetone, followed by three sequential washes with 100 mL of deionized water for five minutes each, employing ultrasonic equipment. Subsequently, the samples were frozen at −53°C for one hour in an ultra-low-temperature freezer and then freeze-dried to remove residual moisture. The dried samples were weighed on an analytical balance with an accuracy of ± 0.5 g. For digestion, each sample was treated with nitric acid in a 10 mL vessel, following a modified version of the EPA 3052 method in an analytical microwave system (Multiwave 3001^™^). The digestion protocol included a five-minute ramp to 190°C, a 20-minute hold at this temperature, and a 20-minute cooling phase. After digestion, the solutions were filtered and diluted with reagent-grade water to a final volume of 50 mL using a volumetric flask (US EPA 2015; Matínez Arroyo 2018).

Trace element analysis of the hair samples was performed using an inductively coupled plasma mass spectrometer (ICP-MS, Thermo Scientific iCAP Q ICP-MS^™^), allowed enabled detection of silver (Ag), arsenic (As), cadmium (Cd), cobalt (Co), chromium (Cr), copper (Cu), mercury (Hg), manganese (Mn), molybdenum (Mo), nickel (Ni), lead (Pb), antimony (Sb), titanium (Ti), and vanadium (V) in the hair samples. For Hg specifically, an inductively coupled plasma optical emission spectrometer equipped with a hydride generation system (ICP-OES, Thermo Scientific iCAP 6500 Duo^™^) was used. The Hg determination involved a hydride generation technique using dilute hydrochloric acid and sodium borohydride, which produced volatile mercury hydrides that were then transported to the plasma for detection. All ICP-MS analyses were performed in Kinetic Energy Discrimination mode, utilizing argon and helium gases to minimize interferences. During analysis, liquid samples were nebulized and carried by argon gas into the plasma torch, generating ions that were subsequently transmitted to the mass spectrometer through an interface. The ions created in the plasma were separated based on their mass-to-charge ratio and quantified with an electron multiplier. Throughout the process, potential confounding factors, including background levels, gas ion contributions, chemical reactions, and matrix effects, were carefully controlled to ensure accuracy and precision (US EPA 2015; Matínez Arroyo 2018).

Limits of detection (LOD) and quantification (LOQ) were determined for each metal/metalloid. The LOD indicates the minimum concentration at which an analyte can be reliably detected above background noise, while the LOQ corresponds to the lowest concentration at which the analyte can be accurately and unbiasedly quantified. This study used the LOQ as the minimum threshold for identifying metals/metalloids in hair samples (Armbruster and Pry 2008). Given the lack of universally accepted reference ranges, upper limits were established based on previous research in healthy populations, excluding values above the 99th percentile, as these were considered atypical. This approach facilitates the interpretation and comparison of results with previous studies on metal/metalloid exposure (Druyan et al. 1998; Rodushkin and Axelsson 2000; Mikulewicz et al. 2013). The LOD and LOQ values for each metal/metalloid are shown in Table 2.

#### 2.2.3. Hair metal/metalloid quality control

For instrument calibration and resolution verification, a mass spectrometer tuning solution containing representative elements (10 μg/L Ba, Bi, Ce, Co, In, Li, U) was employed. The analytical procedure adhered to EPA Method 6020B and incorporated multiple quality control measures, including the use of a method blank, a spiked matrix, a laboratory control sample, and a duplicate sample within each analytical batch. Three types of blanks were applied: a calibration blank consisting of acidified reagent-grade water to assess potential drift, a method blank prepared by digesting a control matrix free of hair but spiked with the target metals and processed under identical conditions as the samples, and a washing blank consisting of a nitric acid solution used to clean the system. Additionally, an analytical blank was prepared by subjecting an acid solution, free of the target analytes, to the same conditions as the control samples. For internal quality control and calibration, certified reference materials were utilized, including a 1,000 μg/mL Hg standard and Quality Control Standard 27 (QCS-27) at 100 μg/mL (both from High Purity Standards) (US EPA 2015; Matínez Arroyo 2018).

Blanks were considered acceptable when analyte levels were below 50% of the quantification limit. Within each analytical sequence, a blank, a verification standard, and a subsequent blank (BR-SV-BR) were analyzed after every ten samples or at the conclusion of the batch. Verification standard recoveries were required to remain within ± 20%; however, higher recoveries were accepted provided that the integrity of the batch was not compromised. Additionally, an analytical blank prepared with the same acid matrix and calibration curve standards was included for every twenty samples. In cases where the verification criterion was not met (i.e., recovery exceeded 20%), the standards were reprocessed, the instrument was recalibrated, and any affected samples were reanalyzed (US EPA 2015; Matínez Arroyo 2018).

Duplicate samples were incorporated into each batch at a frequency of one per twenty samples or per matrix type. Laboratory control samples containing known analyte concentrations were processed concurrently, with acceptance criteria established at ± 20% recovery and ≤ 20% relative percent difference, where the recovery tolerance represents ± 20% of the theoretical value. Matrix spike samples were prepared by fortifying duplicate samples with known metal concentrations before subjecting them to the same digestion and analytical procedures as the original samples. These duplicated and fortified samples were analyzed to evaluate matrix effects, analytical precision, and potential bias, with implementation dependent upon batch characteristics and matrix availability. Fortification was performed at predetermined levels, typically between the low and medium calibration standards. Quality control acceptance criteria were set at ± 25% for bias and ≤ 20% relative percent difference for precision. For samples lacking historical performance data, the acceptance limits were maintained at ± 25% recovery and ≤ 20% relative percent difference (US EPA 2015; Matínez Arroyo 2018).

### 2.3 Sociodemographic and exposure variables

This study included variables extracted from the demographics, health, and housing sections of the 2018 MHAS questionnaire. A socioeconomic status variable was determined using the Centers for Disease Control and Prevention criteria, which encompassed the number of household assets, household perception of economic situation, and food security status. The index variables were summed, with higher scores indicating better socioeconomic status, and subsequently categorized into three groups: low, middle, and high socioeconomic status (CDC 2023).

### 2.4 Statistical Analysis

The statistical analysis included a total of 2,474 participants. Sociodemographic characteristics were described, and the median, interquartile range (IQR), frequency, and percentage were reported. The median, IQR, and minimum and maximum values of Ag, As, Cd, Co, Cr, Cu, Hg, Mn, Mo, Ni, Pb, Sb, Ti, and V concentrations in hair were reported. Hair metal/metalloid concentrations were analyzed for outliers and normal distribution through a histogram, boxplot, quantile-quantile plot, and the Shapiro-Wilks test. Comparisons between metal/metalloid concentrations in hair and sociodemographic characteristics were performed using the χ^2^ test. Statistical analyses were considered significant when the p-value was less than 0.05. Analyses were performed using Jamovi statistical software version 2.7.6.

## 3. Results

### 3.1 Sociodemographic and exposure characteristics

Table 1 presents the sociodemographic characteristics of the study participants. The sample had a median age of 63 years, with a majority of females (58.8%). Concerning marital status, 67.1% of participants were either married or in a consensual union. Educational achievement was predominantly low, as 61.2% of participants had completed between zero and six years of schooling. Additionally, 70.1% resided in suburban or urban areas with populations of 15,000 or more. Notably, 42.5% of the participants belonged to the middle socioeconomic status group.

### 3.2 Hair metal / metalloid concentrations

Table 2 presents the findings regarding the concentrations of twelve metals and two metalloids detected in hair samples, as determined by ICP analysis. The predominant elements detected were Pb (97%), Ti (96%), Mn (96%), and Cu (95%). Conversely, Sb was the least frequently observed, present in merely 4% of samples, followed by Cd (15%), As (24%), and Co (25%). In terms of concentration levels, Cu exhibited the highest median value at 7.83 μg/g (IQR: 5.67–11.20 μg/g), with Ti recorded at 3.42 μg/g (IQR: 2.10–5.36 μg/g). The lowest median concentrations were observed for As and Co (both 0.04 μg/g), Mo (0.07 μg/g), and Cd (0.08 μg/g). Considerable variability was noted in the maximum values of metals detected in hair, with Cu reaching up to 90.70 μg/g, followed by Ti (29.50 μg/g), Ag (28.00 μg/g), and Pb (19.40 μg/g). Significantly, the LOD and the LOQ ranged from 0.003 to 0.013 μg/g and 0.01 to 0.04 μg/g, respectively.

### 3.1 Hair metal/metalloid concentrations and sociodemographic characteristics

Tables 3 to 8 illustrate the variations in hair metal/metalloid concentrations across different sociodemographic characteristics of the participants. The sex-specific analysis (Table 3) identified disparities in the concentrations of thirteen metals and metalloids within hair samples. Relative to females, males demonstrated higher median concentrations of Ag (0.13 vs. 0.10 μg/g), As (0.06 vs. 0.04 μg/g), Cd (0.14 vs. 0.07 μg/g), Co (0.05 vs. 0.03 μg/g), Cr (0.33 vs. 0.14 μg/g), Hg (0.16 vs. 0.13 μg/g), Mn (0.89 vs. 0.73 μg/g), Mo (0.10 vs. 0.06 μg/g), Ni (0.83 vs. 0.52 μg/g), Pb (0.78 vs. 0.59 μg/g), and Sb (0.22 vs. 0.06 μg/g). Conversely, females exhibited higher median concentrations of Cu (8.12 vs. 7.37 μg/g) and Ti (3.81 vs. 2.87 μg/g). V was the sole metal for which no significant sex-related differences in concentration were identified.

Age-related differences were identified in nine of the fourteen metals/metalloids analyzed. Concentrations of Cu, Mn, Ni, Ti, and V generally decreased with advancing age. For example, Ti median concentrations decreased from 3.75 μg/g in the 50–56-year age group to 2.87 μg/g in the 73–99-year age group, while Cu decreased from 8.29 μg/g to 6.85 μg/g across the same age groups. Mn concentrations peaked in the 57–63 age group (1.01 μg/g) and were at their lowest in the 73–99 years (0.58 μg/g). Sb showed variations between age groups, reaching its highest median concentration in the 64–72-year group (0.15 μg/g). Additionally, Pb and Hg concentrations differed among age groups, although no consistent pattern was evident across age ranges. The highest median concentration of Ag was noted in the 64–99-year age group (0.11 μg/g) (Table 4).

The size of the residential locality demonstrated minimal influence on the concentrations of hair metals/metalloids (Table 5). The only element exhibiting variation was Mn, which showed marginally elevated concentrations in urban and suburban localities (0.80 μg/g) relative to rural and semi-rural localities (0.74 μg/g). For the other thirteen metals, no differences were identified between residents of rural/semi-rural and suburban/urban areas.

Hair metal/metalloid concentrations exhibited variability based on the years of education among nine of the fourteen elements analyzed. Individuals with greater educational attainment (≥7 years of schooling) demonstrated higher median hair concentrations of Ag (0.13 vs. 0.09 μg/g), Co (0.04 vs. 0.03 μg/g), Cr (0.21 vs. 0.18 μg/g), Cu (8.73 vs. 7.26 μg/g), Hg (0.17 vs. 0.12 μg/g), Ni (0.67 vs. 0.56 μg/g), Ti (3.58 vs. 3.32 μg/g), and V (0.14 vs. 0.12 μg/g) in comparison to participants with zero to six years of education. In contrast, Mn was the only metal with higher concentrations in the group with lower educational attainment (0.82 vs. 0.75 μg/g) (Table 6).

Marital status was associated with differences in the concentrations of four out of fourteen analyzed metals/metalloids (Table 7). Participants who were married or in a consensual union exhibited higher median concentrations of Cr (0.20 vs. 0.18 μg/g), Mn (0.82 vs. 0.74 μg/g), Ni (0.64 vs. 0.56 μg/g), and Pb (0.71 vs. 0.57 μg/g) compared to those who were single, divorced, separated, or widowed.

Socioeconomic status was associated with differences in the concentrations of multiple metals/metalloids in hair (Table 8). Participants in the middle and high socioeconomic groups exhibited higher median concentrations of Ag (0.12 and 0.10 μg/g, respectively), Cd (0.09 and 0.08 μg/g), Cr (0.20 and 0.19 μg/g), Cu (7.78 and 8.41 μg/g), Hg (0.15 and 0.16 μg/g), Ni (0.61 and 0.60 μg/g), and Pb (0.64 and 0.63 μg/g) compared to those with low socioeconomic status. For Ti, the median concentration was highest in the low socioeconomic group (3.48 μg/g), followed by the high socioeconomic group (3.46 μg/g) and the middle socioeconomic group (3.41 μg/g). V concentrations were also slightly higher in the middle and high categories (0.13 and 0.14 μg/g) than in the low group. Conversely, Mn exhibited the highest concentrations within the low socioeconomic group (0.93 μg/g).

## 4. Discussion

The study reveals extensive exposure to metals/metalloids among older Mexican adults, with Pb, Ti, Mn, and Cu in 95–97% of hair samples. Cu showed the highest median concentration. Significant sex differences were observed: males had higher levels of Ag, As, Cd, Co, Cr, Hg, Mn, Mo, Ni, Pb, and Sb, while females had higher concentrations of Cu and Ti. Age showed different concentrations of Ag, Cu, Mn, Ni, Ti, and V, though Pb and Hg also varied with age, but without a consistent pattern. Locality size had minimal effects, except for slightly higher Mn concentrations in urban/suburban areas. Higher educational attainment (≥ 7 years) was related to increased concentrations of Ag, Co, Cr, Cu, Hg, Ni, Ti, and V, while Mn was elevated in those with lower education. We observed differences in Cr, Mn, Ni, and Pb concentrations depending on the marital status. Socioeconomic status influenced exposure: middleand high-socioeconomic groups had increased levels of Ag, Cd, Cr, Cu, Hg, Ni, Pb, and V, while the lowsocioeconomic group had higher concentrations of Ti and Mn.

Hair analysis serves as a biomarker for metal/metalloid exposure, offering advantages over other methods. It reflects chronic exposure over 1–2 months, making it ideal for assessing metal accumulation. ICP-MS ensures high precision and sensitivity, enabling the detection of low concentrations of metals like As, Cd, Cr, Cu, Hg, Mn, Ni, and Pb. This technique exceeds traditional detection limits, making it valuable for epidemiological and environmental studies (Tobin 2007).

In our study, hair As concentrations (P50: 0.04 μg/g, n 594) are four times higher than those reported in 34 Italian adults aged 55–76 years (P50: 0.01 μg/g) and comparable to those of Russian females aged 50–59 years (P50: 0.02 μg/g, n 21), but lower than in Russian males from the same group (P50: 0.10 μg/g, n 36) (Skalnaya et al. 2016; Schipani et al. 2021). Our findings are also lower than those observed in Shenzhen residents aged 51–60, with 0.07 μg/g in females (n 20) and 0.09 μg/g in males (n 11) (Qin et al. 2021). Moreover, our As concentrations are less than those in various Chinese populations: 0.10 μg/g (P50) in 235 healthy adults from Shaanxi (Yu et al. 2017), 0.20 μg/g (P50) in 1,235 adults from Hunan, median age 55 years (Wang et al. 2021), and 0.30 μg/g (P50) in 35 residents from eleven cities aged 51–65 years (Zhou et al. 2016). Our values are considerably lower than the 1.03 μg/g (P50) found in an elderly Turkish population (${\overset{‾}{x}}_{\text{age}}$: 73, n 40) (Koseoglu et al. 2021).

Cd median concentrations in this study were 0.08 μg/g (n 359). Our concentrations are eight times higher than the 0.01 μg/g median in 34 Italian adults aged 63 years (Schipani et al. 2021), and four times higher than the 0.02 μg/g found in 21 Russian females aged 50–59, yet significantly lower than the 0.52 μg/g in 34 Russian males of the same age (Skalnaya et al. 2016). Our findings slightly exceed the 0.05 μg/g (P50) reported in 255 centenarians from Hainan, China (Hao et al. 2016), and are close to the mean concentrations of 0.07 μg/g in 35 Chinese individuals aged 51–65 (Zhou et al. 2016). However, our Cd concentrations are considerably lower than those in Shenzhen, China, where adults aged 51–60 had concentrations of 0.29 μg/g in 20 females and 0.25 μg/g in 11 males (Qin et al. 2021).

Our study indicates higher Co concentrations in hair (P50: 0.04 μg/g, n 609) compared to the Russian older adult population, where median concentrations were 0.03 μg/g in females (n 21) and males (n 36) aged 50–59 (Skalnaya et al. 2016).

The Cr concentrations (P50: 0.19 μg/g, n 1,922) in our study exceed those in Italian persons (P50: 0.07 μg/g, P50_age_: 63, n 34) but are lower than Russian males (P50: 0.80 μg/g, n 36) and females (P50: 0.28 μg/g, n 21) aged 50–59, and Korean adults (P50: 0.40 μg/g, ${\overset{‾}{x}}_{\text{age}}$: 49; n 207) (Skalnaya et al. 2016; Schipani et al. 2021; Lee et al. 2022). They are closest to the Chinese participants over 80 (P50: 0.29 μg/g, n 152) but still slightly lower than the mean concentrations in Shenzhen residents aged 51–60 (females: n 20, 0.39 μg/g; males: n 11, 0.53 μg/g) (Qin et al. 2021; Zhu et al. 2023). Our results contrast sharply with the high concentrations found in Chinese centenarians (P50: 3.68 μg/g, n 255), which are 19 times higher than ours (Hao et al. 2016).

Cu concentrations in hair from our study (n 2,347, P50: 7.83 μg/g) are similar to those in adults over 80 years (P50: 7.13 μg/g)(Zhu et al. 2023) and 255 Chinese centenarians (P50: 8.09 μg/g) (Hao et al. 2016). However, these results are lower than those of other studies. In Turkey, higher concentrations ($\overset{‾}{x}$: 10.3 μg/g) were found in 31 participants, averaging 73 years (Koç et al. 2015), while older adults (n 10, ${\overset{‾}{x}}_{\text{age}}$: 74) in Estarreja, Portugal, had concentrations of 10.57 μg/g (Cabral Pinto et al. 2019). Russians aged 50–59 showed 15.31 μg/g (P50) in females (n 21) and 12.62 μg/g (P50) in males (n 36) (Skalnaya et al. 2016), while residents of Shenzhen, China (ages 51–60) had average Cu concentrations of 14.51 μg/g in females (n 20) and 13.69 μg/g in males (n 11) (Qin et al. 2021). Notably, a study of 31 adults with a mean age of 63 reported mean concentrations of 18.10 μg/g (Król et al. 2019); the most considerable differences were observed in a study of 207 Korean adults, with median concentrations of 19.0 μg/g (Lee et al. 2022).

Our study of hair Hg concentrations in 2,108 participants found median concentrations of 0.14 μg/g, similar to those in central Poland 0.15 μg/g (P50) in adults aged 46–66 years (n 60) (Marcinek-Jacel et al. 2017). However, our results are lower than those in Russian adults aged 50–59 years, where females (n 21) had 0.51 μg/g (P50) and males (n 36) 1.16 μg/g (P50) (Skalnaya et al. 2016). Our findings also fall short of a Portuguese study in older adults (n 10, ${\overset{‾}{x}}_{\text{age}}$: 74), which reported a mean hair Hg concentration of 0.88 μg/g (Cabral Pinto et al. 2019). Higher concentrations were also documented in Shenzhen, China, among residents aged 51–60 years, with averages of 0.53 μg/g in females (n 20) and 0.69 μg/g in males (n 11) (Qin et al. 2021).

In this study, the median Mn hair concentration was 0.79 μg/g (n 2,371), a value close to that reported in Russian adult males aged 50–59 years, who showed a median of 0.82 μg/g (n 36), and slightly lower than that found in Russian females of the same age, with 1.08 μg/g (n 21) (Skalnaya et al. 2016). Similar results to the latter were observed in Polish adults ($\overset{‾}{x}$: 1.22 μg/g; ${\overset{‾}{x}}_{\text{age}}$: 49.3 years; n 60) and in Taiwanese participants ($\overset{‾}{x}$: 1.19 μg/g; ${\overset{‾}{x}}_{\text{age}}$: 53.1 years; n 60) (Błażewicz et al. 2017). Values tended to increase more notably in older populations in China, where elderly and centenarians (${\overset{‾}{x}}_{\text{age}}$: 92 years; n152) reached a median of 1.85 μg/g (Zhu et al. 2023), while centenarians from Hainan Province (n 255) showed an even higher median of 1.90 μg/g (Hao et al. 2016). Even higher values were recorded in Chinese adults aged 51–60 years, with means of 2.30 μg/g in females (n 20) and 2.99 μg/g in males (n 11) (Qin et al. 2021). On the other hand, lower concentrations were reported in older groups from Poland ($\overset{‾}{x}$: 0.4 μg/g; ${\overset{‾}{x}}_{\text{age}}$: 73.18 years; n 33) (Koç et al. 2015), in Portuguese subjects with a mean age of 74 years ($\overset{‾}{x}$: 0.19 μg/g; n 10) (Cabral Pinto et al. 2019), and particularly in middle-aged Koreans (38–60 years), with a mean of only 0.18 μg/g (n 207) (Lee et al. 2022).

The median Ni concentration in our study’s hair samples (n 2,104) was 0.60 μg/g. This aligns with Russian males aged 50–59 years (n 36, P50: 0.57 μg/g) but is higher than in females (n 21, P50: 0.41 μg/g) (Skalnaya et al. 2016). Our concentrations are lower than in healthy centenarians (n 255) from Hainan, China, where the median Ni was 0.96 μg/g (Hao et al. 2016). However, our values exceed those in an Italian population (${\overset{‾}{x}}_{\text{age}}$: 63, n 34), with a median concentration of 0.11 μg/g (Schipani et al. 2021). Notably, our findings are also higher than those of a Shenzhen study of residents aged 51–60, which reported average Ni concentrations of 0.30 μg/g in females (n 20) and 0.37 μg/g in males (n 11) (Qin et al. 2021).

Our study’s Pb concentrations (n 2,404) showed a median concentration of 0.65 μg/g in hair samples, significantly lower than the 3.81 μg/g mean in 35 Chinese adults aged 51–65 years (Zhou et al. 2016). Comparatively, a median of 1.77 μg/g was reported in 152 Chinese adults over 80 years (Zhu et al. 2023) and 1.24 μg/g in 255 centenarians from Hainan (Hao et al. 2016). Shenzhen residents aged 51–60 exhibited notably higher concentrations, with mean concentrations of 7.40 μg/g in females (n 20) and 8.20 μg/g in males (n 11) (Qin et al. 2021). Our results align more with European findings, slightly exceeding the median concentration of 0.44 μg/g in 34 Italians with a median age of 63 years (Schipani et al. 2021), and the mean level of 0.29 μg/g found in 10 Portuguese residents with a mean age of 74 years from Estarreja (Cabral Pinto et al. 2019). Compared to the Orenburg study of adults aged 50–59, our median values fell between the reported 0.34 μg/g for females (n 21) and 3.02 μg/g for males (n 36) (Skalnaya et al. 2016).

This study found median V concentrations in hair of 0.13 μg/g in 1,525 participants, exceeding the median concentrations in a study of the Russian population aged 50 to 59, which were 0.12 μg/g (n 36) for males and 0.06μg/g (n 21) for females (Skalnaya et al. 2016).

In the past decade, few scientific studies have reported Ag, Mo, Sb, and Ti concentrations in hair samples from individuals aged 50 and older. Although various elements have been analyzed as biomarkers in human hair, no studies have focused on these four metals in this age group, thereby restricting our understanding of their body burden and potential health effects.

Our study offers a significant methodological advantage with much larger sample sizes than those reported in metal/metalloid biomonitoring studies. Unlike most published research, which often relies on small samples—oftentimes fewer than 100 participants—our research encompasses much larger populations across all measured elements. Even compared to more extensive studies, such as in Hunan, China (n 1,235)(Wang et al. 2021) or the Chinese centenarian study (n 255) (Hao et al. 2016), our sample sizes for most elements are still substantially greater. This enables more precise conclusions about exposure patterns and reduces individual variability, resulting in a more comprehensive assessment of metal/metalloid exposure in our target population.

We observed sex differences in hair metal concentrations, with males exhibiting higher concentrations of certain metals/metalloids than females. Sex affects metal accumulation and distribution in the body through biological and sociocultural factors. Biological aspects include hormonal variations, where estrogen offers protection, body composition differences, and sex-specific gene expression that affects metal/metalloid metabolism (Pamphlett et al. 2018; Wai et al. 2020; Gade et al. 2021; Huang et al. 2022; Li et al. 2024). Sociocultural factors encompass occupational exposures and habits, such as females using cosmetics with trace metals and males consuming more tobacco and alcohol (Macías Ruiz et al. 2023; Song et al. 2023). These differences underscore the importance of sex-specific risk assessments and tailored prevention strategies to reduce health effects (Lv et al. 2021; Zhang et al. 2023).

This study found a link between age and metal/metalloid concentrations in the sample. Research indicates that metal/metalloid concentrations generally increase with age due to chronic exposure. This can lead to oxidative stress, particularly in older adults. Age also affects the presence of metals/metalloids through factors such as environmental exposure, changes in tissue permeability, bone storage capacity, and the efficiency of detoxification systems. Moreover, comorbidities like hypertension and diabetes, which increase with age, are influenced by metal/metalloid concentrations. This emphasizes the importance of monitoring metal/metalloid exposure in aging populations to prevent associated diseases (Hudgens et al. 2016; Bae et al. 2017; Chang et al. 2018; Pamphlett et al. 2018; Baudry et al. 2020; Tan et al. 2021; Zhang et al. 2021; Barahona et al. 2022; Park and Lee 2022; Xu et al. 2024; Abu Orabi et al. 2024).

Residential locality size minimally influenced hair metal/metalloid concentrations, except for Mn, which was higher in urban and suburban areas. This variation reflects differences in the sources of exposure. In urban settings, industries such as iron-manganese alloy foundries increase the inhalation of airborne Mn particles (Colledge et al. 2015). At the same time, in rural areas, exposure primarily occurs through groundwater that contains naturally occurring Mn, particularly in regions with Mn-rich soils (Coetzee et al. 2016). Agricultural practices, including those using Mn-containing pesticides, also contribute to chronic exposure through resuspended dust and crop contamination (Ruiz-Azcona et al. 2021). Issues such as poorly regulated rural wells and limited water treatment increase rural exposure (Butler et al. 2019), while the dispersion of Mn is affected by wind patterns and industrial density (Friedman et al. 2024).

Hair metal/metalloid concentrations varied by educational attainment for nine of the 14 analyzed elements. Research reveals three ways education influences adult metal/metalloid exposure and bioaccumulation. First, occupational exposure: individuals with less than 12 years of schooling often work manual jobs involving metals/metalloids, such as mining or agriculture with agrochemicals. Second, residing close to sources of contamination is a significant factor; lower educational attainment correlates with living near or in polluted areas. Ultimately, dietary patterns vary according to education level. Higher education is associated with increased antioxidant intake and consumption of anti-inflammatory diets, which in turn reduce intestinal metal/metalloid absorption and oxidative stress. These findings underscore the intricate relationship between education and metal/metalloid exposure, underscoring the importance of addressing educational disparities in public health strategies (Laouali et al. 2022; Sasaki and Carpenter 2022; Li et al. 2022; Kuo et al. 2024).

Marital status influenced concentrations of four of 14 metals/metalloids. Behavioral and environmental factors mediate this relationship. Household exposures linked to marital status may influence the absorption of certain metals. Married or cohabiting individuals often have more metabolic disorders, possibly due to different dietary patterns resulting in elevated metal/metalloid concentrations. Additionally, factors such as stress from dysfunctional relationships or social support in stable marriages can affect metal/metalloid concentrations through behaviors like smoking. Thus, marital status primarily indicates factors that may alter metal/metalloid exposure, and it is not a direct determinant of exposure (Li et al. 2023; Huang et al. 2023; Tang et al. 2024; Yao and Xu 2024).

Recent research highlights how socioeconomic status influences exposure to heavy metals/metalloids in individuals over 50. Studies have shown a link between poverty, minority groups, and higher levels of As, Cd, Cr, Pb, Ba, Se, and Ag in urban soils, which can impact older adults living near industrial sources (Jones et al. 2022). Biomonitoring in regions such as the Americas, China, and Brazil reveals that lower socioeconomic status—characterized by less education, lower income, and inadequate healthcare—leads to a greater accumulation of toxic metals, including Cd, Pb, Ni, Cu, and Hg (Zhao et al. 2019; Bakulski et al. 2020; Campos et al. 2021; Feng et al. 2025). Hair biomarker data indicate that middle- and high-socioeconomic groups may have higher median concentrations of Ag, Cd, Cr, Cu, Hg, Ni, Pb, and V. In contrast, Ti and Mn are more prevalent among the low socioeconomic group, likely due to different exposures. The distribution suggests complex exposure mechanisms from the environment, diet, and genetics (Xiao et al. 2022; Ye et al. 2024). A consistent gradient in health risk—such as kidney, brain, heart, and functional issues—highlights socioeconomic status as a key factor in population health. These findings underscore the need for policies that reduce environmental injustice and protect vulnerable older adults through targeted surveillance and interventions that are accessible to all socioeconomic levels (Campos et al. 2021; Jones et al. 2022; Zheng et al. 2024; Bakulski et al. 2020; Feng et al. 2025; Ye et al. 2024; Zhao et al. 2019; Xiao et al. 2022).

In addition to its cross-sectional design, this study presents further limitations. First, using hair samples as biomarkers makes results susceptible to exogenous contamination from environmental sources, which can influence concentration estimates despite rigorous washing protocols. Moreover, hair only reflects recent exposures (1–2 months) and may not capture cumulative or long-term exposure patterns. The lack of universally accepted reference ranges for hair concentrations also restricts the validity of direct comparisons.

## 5. Conclusions

This study sheds light on the exposure to metals and metalloids in older Mexican adults, paving the way for future research on their health impacts and potential prevention strategies. Although the concentrations of most elements are comparable to those in other populations, exposure to multiple metals raises concerns about cumulative health effects. Given this population’s vulnerability, it is crucial to continue investigating sources of exposure and potential impacts of these exposures.

## Supplementary Material

Supplementary Files

This is a list of supplementary files associated with this preprint. Click to download.

• Table.docx

## Abbreviations

Ag: Silver
As: Arsenic
Cd: Cadmium
Co: Cobalt
Cr: Chromium
Cu: Copper
Hg: Mercury
ICP-MS: Inductively Coupled Plasma Mass Spectrometer
ICP-OES: Inductively Coupled Plasma Optical Emission Spectroscopy
IQR: Interquartile Range
MHAS: Mexican Health and Aging Study
Mn: Manganese
Mo: Molybdenum
Ni: Nickel
LOD: Limit of Detection
LOQ: Limits of Quanti cation
Pb: Lead
Sb: Antimony
Ti: Titanium
V: Vanadium
